# Sex-Dependent Role of Adipose Tissue HDAC9 in Diet-Induced Obesity and Metabolic Dysfunction

**DOI:** 10.3390/cells11172698

**Published:** 2022-08-30

**Authors:** Brandee Goo, Samah Ahmadieh, Abdalrahman Zarzour, Nicole K. H. Yiew, David Kim, Hong Shi, Jacob Greenway, Stephen Cave, Jenny Nguyen, Swetha Aribindi, Mark Wendolowski, Praneet Veerapaneni, Mourad Ogbi, Weiqin Chen, Yun Lei, Xin-Yun Lu, Ha Won Kim, Neal L. Weintraub

**Affiliations:** 1 Vascular Biology Center, Medical College of Georgia, Augusta University, 1120 15th St., CB3940, Augusta, GA 30912, USA; 2 Department of Medicine, Medical College of Georgia, Augusta University, 1120 15th St., BI5076, Augusta, GA 30912, USA; 3 Departments of Physiology and Regenerative Medicine, Medical College of Georgia, Augusta University, 1120 15th St., CA3126, Augusta, GA 30912, USA; 4 Departments of Neuroscience and Regenerative Medicine, Medical College of Georgia, Augusta University, 1120 15th St., CA3008, Augusta, GA 30912, USA

**Keywords:** HDAC9, obesity, insulin resistance, high fat diet

## Abstract

Obesity is a major risk factor for both metabolic and cardiovascular disease. We reported that, in obese male mice, histone deacetylase 9 (HDAC9) is upregulated in adipose tissues, and global deletion of HDAC9 protected against high fat diet (HFD)-induced obesity and metabolic disease. Here, we investigated the impact of adipocyte-specific *HDAC9* gene deletion on diet-induced obesity in male and female mice. The *HDAC9* gene expression was increased in adipose tissues of obese male and female mice and HDAC9 expression correlated positively with body mass index in humans. Interestingly, female, but not male, adipocyte-specific *HDAC9* KO mice on HFD exhibited reduced body weight and visceral adipose tissue mass, adipocyte hypertrophy, and improved insulin sensitivity, glucose tolerance and adipogenic differentiation gene expression. Furthermore, adipocyte-specific *HDAC9* gene deletion in female mice improved metabolic health as assessed by whole body energy expenditure, oxygen consumption, and adaptive thermogenesis. Mechanistically, compared to female mice, HFD-fed male mice exhibited preferential *HDAC9* expression in the stromovascular fraction, which may have offset the impact of adipocyte-specific *HDAC9* gene deletion in male mice. These results suggest that HDAC9 expressed in adipocytes is detrimental to obesity in female mice and provides novel evidence of sex-related differences in HDAC9 cellular expression and contribution to obesity-related metabolic disease.

## 1. Introduction

Adipose tissues regulate whole body energy homeostasis and systemic insulin sensitivity. Adipose tissue dysfunction, characterized by adipocyte hypertrophy, insulin resistance, diminished thermogenic capacity, etc., contributes to diet-induced obesity (DIO) and metabolic disease [1]. During chronic caloric excess, adipose tissues can expand via adipocyte hypertrophy or hyperplasia, with the latter considered a metabolically healthier form of adipose tissue expansion. Adipose tissues are comprised of a stromovascular fraction (SVF) and a mature adipocyte fraction (MAF). The SVF contains many preadipocytes, which are partially committed stem cells that can readily differentiate into mature adipocytes capable of efficiently storing lipid. Nevertheless, adipocyte hypertrophy prevails in DIO, leading to worsening metabolic disease, ectopic lipid accumulation, etc. However, the mechanisms that regulate adipocyte hypertrophy during DIO are poorly understood and understanding these mechanisms could lead to new therapeutic targets in obesity.

We have previously demonstrated that histone deacetylase 9 (HDAC9) is a negative regulator of adipogenic differentiation, and its downregulation in preadipocytes in response to adipogenic signals is obligatory for adipogenic differentiation. In male mice, HDAC9 expression is increased in adipose tissues during DIO, resulting in the accumulation of dysfunctional adipocytes. Preadipocytes cultured from high fat diet (HFD)-fed male mice exhibited impaired HDAC9 downregulation and poor adipogenic differentiation potential. Moreover, global *HDAC9* gene deletion protected male mice against DIO and improved adipogenic differentiation and *HDAC9* gene deletion reduced insulin resistance, adipocyte hypertrophy and ectopic lipid deposition. Importantly, global *HDAC9* gene deletion enhanced whole body energy expenditure and adaptive thermogenesis, suggesting that it regulates the balance between caloric storage and caloric combustion in adipose tissues. However, HDAC9 is ubiquitously expressed and our prior studies were exclusively conducted in male mice. Thus, the role of adipocyte-specific HDAC9 expression in DIO in male versus female mice remains to be determined.

Here, we investigated the effect of adipocyte-specific *HDAC9* gene knockout (A-KO) on adipose tissue function and metabolic disease in the setting of DIO. We observed that HFD-feeding increased expression of *HDAC9* in adipose tissues of mice and HDAC9 expression correlated positively with body mass index (BMI) in humans. Female A-KO mice were partially protected against HFD-induced weight gain and adipocyte hypertrophy while showing elevated energy expenditure, expression of metabolic and beiging genes and adaptive thermogenesis. Unexpectedly, adipose-specific *HDAC9* gene deletion had no impact on DIO in male mice. Mechanistically, compared to female mice, male mice had much higher *HDAC9* expression in the SVF of adipose tissues under HFD-fed conditions. Our findings provide evidence that adipocyte-expressed HDAC9 contributes to HFD-induced obesity only in female mice and suggest that cellular HDAC9 expression is regulated in a sex-dependent manner in adipose tissues.

## 2. Materials and Methods

### 2.1. Mice

The *HDAC9* floxed mice were generated by flanking the loxP site in the promoter region and the exon 4 of *HDAC9*. To generate adipocyte-specific *HDAC9* KO mice, *HDAC9* floxed (*HDAC9*^flox/flox^) mice in the C57BL/6 background were crossed with adiponectin-Cre mice (C57BL/6J background, Jackson Laboratories, Bar Harbor, USA), which express Cre recombinase in mature adipocytes. Briefly, male adiponectin-Cre hemizygous *HDAC9*^flox/-^ offspring were subsequently crossed with female *HDAC9*^flox/flox^ mice to obtain *HDAC9*^flox/flox^/adiponectin-Cre^+/−^ mice. Then, male *HDAC9*^flox/flox^/adiponectin-Cre^+/−^ mice were subsequently crossed with female *HDAC9*^flox/flox^ mice to generate *HDAC9*^flox/flox^ mice (hereafter referred to as WT) and *HDAC9*^flox/flox^/adiponectin-Cre^+/−^ mice (A-KO). Mice were maintained on a chow diet (CD) after weaning. At 8–14 weeks, mice were maintained on CD (Harlan Teklad, LM-485) or switched to HFD (Research Diet, D12492, 60% calories from fat) and housed at thermoneutral temperature (27.5–30 °C) for up to ~14 months. Food and water were provided ad libitum. Mice were weighed weekly. Following 40–60 weeks HFD feeding, mice were perfused with ice-cold saline and tissues were harvested and weighed. All animal studies were conducted using a protocol approved by the Institutional Animal Care and Use Committee of Augusta University following appropriate guidelines.

### 2.2. Human Subjects

Discarded human subcutaneous adipose tissue was collected from patients undergoing cardiothoracic surgery in operating rooms of Augusta University Health. Investigations in this study were carried out in accordance with the rules of the Declaration of Helsinki of 1975.

### 2.3. Adipose Tissue Fractionation

Adipose tissue was separated into the SVF and MAF as previously described [2,3,4,5,6]. Briefly, subcutaneous (SQ) and gonadal visceral (VF) adipose tissue was excised, minced, and digested with type I collagenase. The SVF and MAF tissues were separated by centrifugation and washed with saline.

### 2.4. Quantitative PCR

Total RNA was extracted from whole adipose tissue samples or fractions with Qiazol and processed using with RNeasy Lipid Tissue Mini Kit (Qiagen, Hilden, Germany). The RT-PCR quantification of mRNA levels was performed using SYBR green qRT-PCR kits (Agilent Technologies, Santa Clara, USA; abm, Richmond, Canada). Primer sequences are listed in Table S2. Fold change was calculated by ΔΔCt method.

### 2.5. Western Blot

Protein was extracted from adipose tissues using a Tissue-Tearor (BioSpec, Bartlesville, USA) in RIPA buffer with protease inhibitors, followed by centrifugation, separation by SDS-PAGE, transfer to nitrocellulose membrane and probing with appropriate antibody. Blots were developed with ECL system. Antibodies were obtained from Biorbyt, Cambridge, United Kingdom (HDAC9; orb214926), Invitrogen, Waltham, USA (GAPDH; AM4300) and BD Biosciences, San Jose, USA (HSP90; 610418).

### 2.6. Locomotor Activity, Food Consumption and Energy Expenditure Measurements

Locomotor activity, food consumption and energy expenditure were measured in 9 to 10-month-old mice using a comprehensive laboratory animal monitoring system (CLAMS, Columbus Instruments, Columbus, USA) for 3 days (24 h acclimation followed by 48 h measurement) as previously reported [3,7,8].

### 2.7. Body Composition Measurements

Fat and lean mass were measured in approximately 40-week-old mice using nuclear magnetic resonance (NMR) spectroscopy (Bruker Minispec LF90II, Bruker, Billerica, USA) as previously reported and normalized to total body weight [7,8].

### 2.8. Hepatic Triglyceride Measurement

Flash frozen liver samples were briefly homogenized in isopropyl alcohol at 50 mg/mL using a Tissue-Tearor (Biospec, Bartlesville, USA). Triglyceride concentration was measured with LabAssay™ Triglyceride Kit (FujiFilm Healthcare, Lexington, MA, USA) as previously described and normalized to liver sample mass.

### 2.9. Adaptive Thermogenesis

At approximately 14 to 16 months of age (12–14 months HFD feeding), mice were subjected to 4 °C for 3 h in the absence of food using an environmental chamber (Powers Scientific, Inc., Doylestown, USA) Rectal temperature was measured at baseline and at 1, 2 and 3 h following placement in the environmental chamber.

### 2.10. Histology and Measurement of Adipocyte Cell Size

Adipose tissues were fixed in 10% formalin, dehydrated in ethanol, transferred to xylene solution, and embedded in paraffin. Tissue sections were stained in hematoxylin and eosin (H&E). Images were analyzed using Adiposoft plugin (Pamplona, Spain) for Fiji [9] to quantify adipocyte size as previously described [3].

### 2.11. Glucose Tolerance Test

At approximately 11–12 months of age (9–11 months HFD feeding), mice were fasted for 12 h followed by intraperitoneal injection of glucose at 2 g/kg body weight. Glucose levels were measured via tail vein at baseline and every 10 min up to 2 h following glucose injection as previously described [3,7,8].

### 2.12. Insulin Tolerance Test

At approximately 11–12 months of age (9–11 months HFD feeding), mice were fasted for 6 h followed by intraperitoneal injection with regular insulin (Humulin) at 0.75 units/kg body weight. Glucose levels were measured at baseline and every 10 min up to 90 min as described above [3,7,8].

### 2.13. Statistics

GraphPad Prism version 9 software (GraphPad Software, Inc., San Diego, USA) was used for statistical calculations. Data are expressed as mean ± SEM (standard error of the mean). The Shapiro–Wilk test was used to test the normality. The *F* test was used to test for equality of variance. Unpaired two-tailed *t*-tests were used to compare between two groups with equal variances. Welch’s correction was used for two-sample comparison between groups with unequal variances. Two-way analysis of variance followed by Tukey post hoc tests were used for comparisons between groups. Simple linear regression was used for correlation analysis. *p* < 0.05 was considered significant.

## 3. Results

### 3.1. Elevated Expression of HDAC9 under Obese Conditions

**Figure 1:**
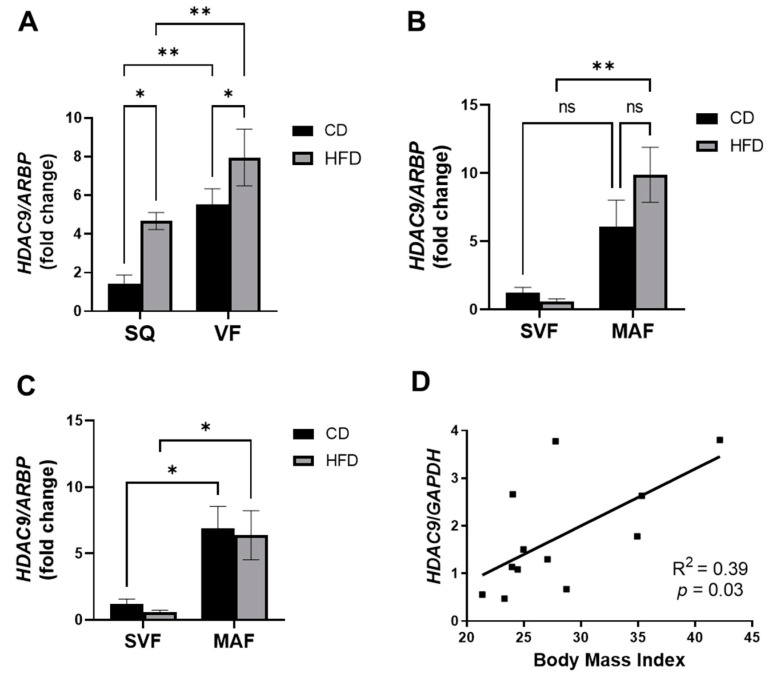
*HDAC9* expression is increased in adipose tissue under obese-conditions. (**A**) *HDAC9* mRNA expression in SQ and VF from CD- and HFD-fed female WT mice. *HDAC9* mRNA expression in SVF and MAF from SQ (**B**) and VF (**C**) of female CD- and HFD-fed WT mice (*n* = 5–6). (**D**) HDAC9 protein expression in human SQ correlated with body mass index (*n* = 12). Data represent mean ± SEM. * *p* < 0.05, ** *p* < 0.01, ns, not significant. SQ, subcutaneous adipose tissue; VF, visceral adipose tissue; CD, chow diet; HFD, high fat diet; SVF, stromovascular fraction; MAF, mature adipocyte fraction.

First, we evaluated the effect of HFD on *HDAC9* gene expression in SQ and VF adipose tissue from female mice. Consistent with our previous findings in male mice, HFD significantly increased adipose tissue *HDAC9* expression in SQ and VF adipose tissue of female mice (Figure 1A). We previously reported that in human adipose tissues from non-obese non-diabetic patients, *HDAC9* expression is higher in VF compared to SQ adipose tissue [2]. Similarly, *HDAC9* expression was higher in VF compared to SQ adipose tissues in female mice (Figure 1A).

We have previously demonstrated that HDAC9 is downregulated during adipogenic differentiation in preadipocytes from CD-fed mice, and that this downregulation is abolished in preadipocytes from HFD-fed mice. To further dissect the expression of HDAC9 in adipose tissue, we fractionated adipose tissues from HFD-fed mice (~1 year of age) into the SVF, which primarily contains preadipocytes, endothelial cells and immune cells, and the MAF. Successful fractionation was confirmed by demonstrating markedly higher *adiponectin* gene expression in the MAF compared to the SVF of both adipose depots (Figure S1). Interestingly, *HDAC9* expression was significantly higher in the MAF compared to the SVF in both depots of CD- and HFD-fed female mice (Figure 1B,C).

To examine the potential association of HDAC9 with human obesity, we measured HDAC9 expression in human subcutaneous adipose tissue. Interestingly, HDAC9 expression positively correlated with body mass index (BMI, Figure 1D). Demographic data are shown in Table S1. These data suggest that adipocyte HDAC9 may also play a role in human adipose tissue dysfunction in obesity.

### 3.2. Generation of Adipocyte-Specific HDAC9 KO Mice

**Figure 2:**
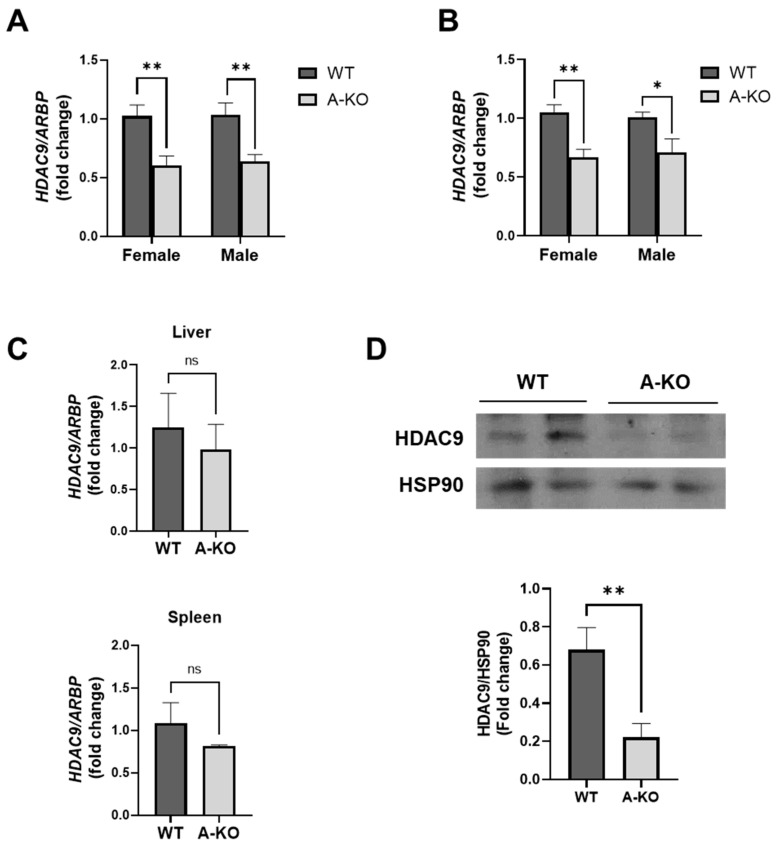
Generation of adipocyte-specific *HDAC9* KO mice. *HDAC9* mRNA expression in SQ (**A**) and VF (**B**) from male and female HFD-fed mice (*n* = 5–9). (**C**) *HDAC9* mRNA expression level in tissues from female WT and A-KO fed a HFD (*n* = 4). (**D**) Western blot validation of HDAC9 protein expression in VF from HFD-fed female mice (*n* = 6). Data represent mean ± SEM. * *p* < 0.05, ** *p* < 0.01, ns, not significant. A-KO, adipocyte-specific *HDAC9* KO; SQ, subcutaneous adipose tissue; VF, visceral adipose tissue.

We previously demonstrated that HFD-feeding impairs the downregulation of HDAC9 during adipogenic differentiation, likely leading to the accumulation of inefficiently differentiated adipocytes [7]. Therefore, to examine the role of adipocyte-expressed HDAC9 in HFD-induced obesity and metabolic disease, *HDAC9* floxed mice were bred with adiponectin-cre mice to generate adipocyte-specific *HDAC9* knockout mice (A-KO) on a C57BL/6 background. Downregulation of *HDAC9* in A-KO mice was validated by RT-PCR in SQ (Figure 2A) and VF adipose tissues (Figure 2B) from male and female mice following HFD feeding. Liver and spleen *HDAC9* mRNA expression was unaffected, confirming selective deletion of *HDAC9* expression in adipose tissues (Figure 2C). We also confirmed that HDAC9 protein expression was reduced in VF from HFD-fed mice (Figure 2D).

### 3.3. Adipocyte HDAC9 Gene Deletion Ameliorates HFD-Induced Obesity in Female Mice

**Figure 3:**
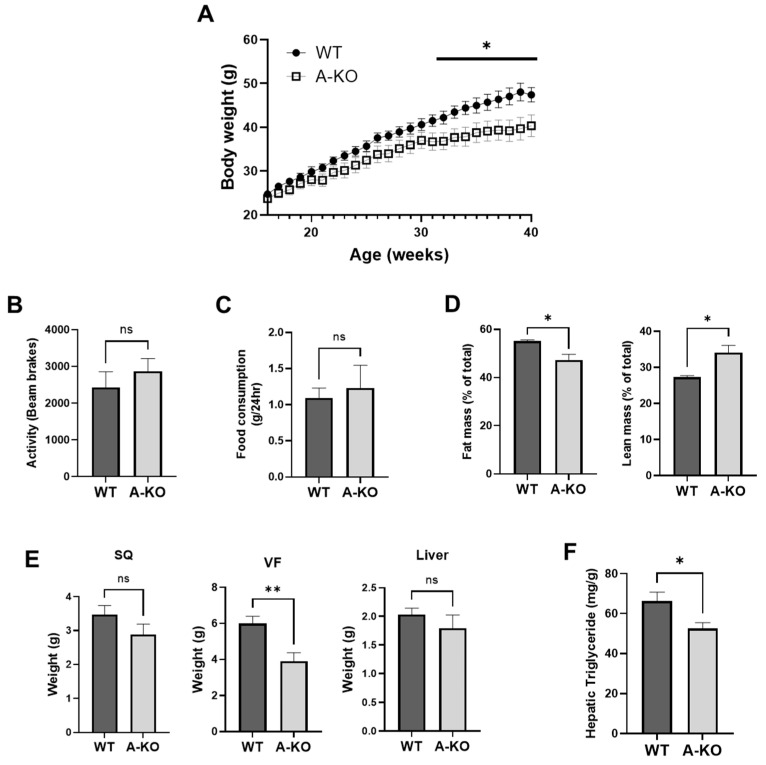
Female A-KO mice have reduced weight gain and fat mass following HFD-feeding. (**A**) Growth curves of female WT and A-KO mice fed a HFD (*n* = 11–15). (**B**) Cumulative locomotor activity over a 24 h period (*n* = 8). (**C**) Cumulative food consumption over a 24 h period (*n* = 7). (**D**) Fat mass and lean mass measured by whole body composition nuclear magnetic resonance (*n* = 11–16). (**E**) Tissue weight measurements of SQ, VF and liver at time of sacrifice (*n* = 10–15). (**F**) Hepatic triglyceride measured per gram liver (*n* = 10–16). Data represent mean ± SEM. * *p* < 0.05, ** *p* < 0.01, ns, not significant. SQ, subcutaneous adipose tissue; VF, visceral adipose tissue.

To determine whether adipose-specific *HDAC9* gene deletion favorably impacts the development of HFD-induced obesity, total body weight was measured weekly in WT and A-KO mice. The HFD-fed female A-KO mice exhibited reduced weight gain (Figure 3A) despite maintaining similar locomotor activity (Figure 3B) and food consumption (Figure 3C) compared to HFD-fed WT mice. As expected, reduced adipose mass was observed in A-KO mice as measured by NMR spectroscopy (Figure 3D). Furthermore, at the time of sacrifice, these mice exhibited significantly lower VF weight, and a trend toward reduced SQ weight, compared to WT mice (Figure 3E). Liver is a site of ectopic lipid deposition in the HFD model of obesity. Interestingly, liver from female HFD-fed A-KO mice had a slight trend toward reduced mass compared to HFD-fed WT mice (Figure 3E), suggesting reduced hepatic steatosis in A-KO mice. Accordingly, triglyceride content was significantly lower in liver samples from A-KO mice compared to WT mice (Figure 3F). Taken together, these data suggest that adipocyte HDAC9 contributes to increased adiposity and ectopic lipid deposition in HFD-fed female mice.

### 3.4. Adipocyte HDAC9 Regulates Energy Expenditure and Adaptive Thermogenesis

**Figure 4:**
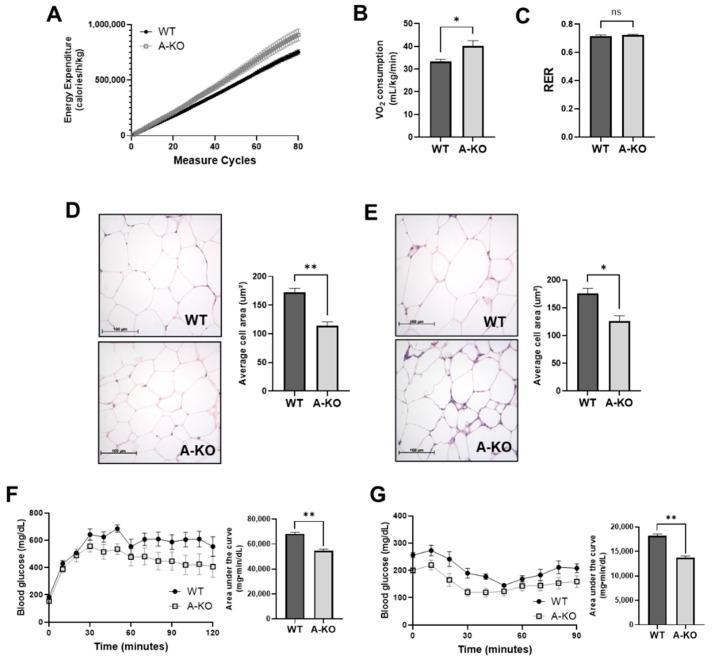
Adipocyte-specific *HDAC9* gene deletion increases energy expenditure and metabolic health in HFD-fed female mice. Comprehensive lab animal monitoring system (CLAMS) measurement of energy expenditure (**A**), VO_2_ consumption (**B**), and respiratory exchange ratio (RER) (**C**) from HFD-fed female mice (*n* = 8). Representative H&E images of SQ (**D**) and VF (**E**) from HFD-fed female WT and A-KO mice with quantification of adipocyte size (*n* = 3). Glucose tolerance (**F**) and insulin tolerance test (**G**) quantified by area under the curve (*n* = 5–7). Data represent mean ± SEM. * *p* < 0.05; ** *p* < 0.01, ns, not significant. SQ, subcutaneous adipose tissue; VF, visceral adipose tissue.

Our data indicate that female HFD-fed A-KO mice exhibit reduced obesity despite similar activity level and food consumption, akin to that reported in global *HDAC9* KO mice, which was attributed to increased energy expenditure. Similarly, HFD-fed female A-KO mice had higher energy expenditure (Figure 4A) and oxygen consumption (Figure 4B) compared to WT mice. Furthermore, no changes in respiratory exchange quotient were identified (Figure 4C), as was observed in global *HDAC9* KO mice. These findings suggest that deletion of *HDAC9* in mature adipocytes promotes combustion of excess calories to combat DIO in female mice.

### 3.5. Adipocyte HDAC9 Gene Deletion Reduces Adipocyte Hypertrophy and Improves Glucose Tolerance and Insulin Sensitivity in HFD-Fed Female Mice

Increased caloric combustion in adipose tissues might lead to diminished adipocyte hypertrophy in DIO. We therefore measured the adipocyte size using H&E staining to quantify adipocyte hypertrophy. Female HFD-fed A-KO mice exhibited reduced adipocyte size in SQ (Figure 4D) and VF (Figure 4E) adipose tissues compared to HFD-fed WT mice.

Next, we examined metabolic status in these mice following approximately 9 months HFD-feeding by performing glucose tolerance (GTT) and insulin tolerance (ITT) testing. During GTT, glucose levels were blunted in A-KO mice compared to WT, as quantified by reduced area under the curve (AUC, Figure 4F). Furthermore, A-KO mice exhibited more robust responses to insulin during ITT (Figure 4G). These data suggest that deletion of *HDAC9* in mature adipocytes improves metabolic phenotype in HFD-fed female mice.

### 3.6. Body Weight and Metabolic Status Is Not Significantly Altered by Adipocyte HDAC9 Deletion in CD-Fed Mice

The A-KO mice fed a CD showed significant reduction of *HDAC9* expression in whole SQ (Figure S2). In contrast to our findings in HFD-fed mice, A-KO female (Figure S2) and male (Figure S3) mice fed a CD did not exhibit alterations in body mass or composition. Similarly, there was no difference in adipocyte size between female A-KO and WT mice fed a CD (Figures S2 and S3). WT and A-KO mice of both sexes likewise had similar responses to glucose or insulin injection during GTT and ITT, respectively (Figures S2 and S3), with a moderately faster return to fasting glucose level in female A-KO mice following insulin injection compared to the WT mice (Figure S2G). These data suggest that adipocyte-expressed HDAC9 does not play a major role in regulating adipose tissue function in lean, healthy states in female or male mice.

### 3.7. Adipocyte HDAC9 Gene Deletion Improves Adaptive Thermogenesis and Upregulates Expression of Adipogenic, Metabolic and Beiging Genes

**Figure 5:**
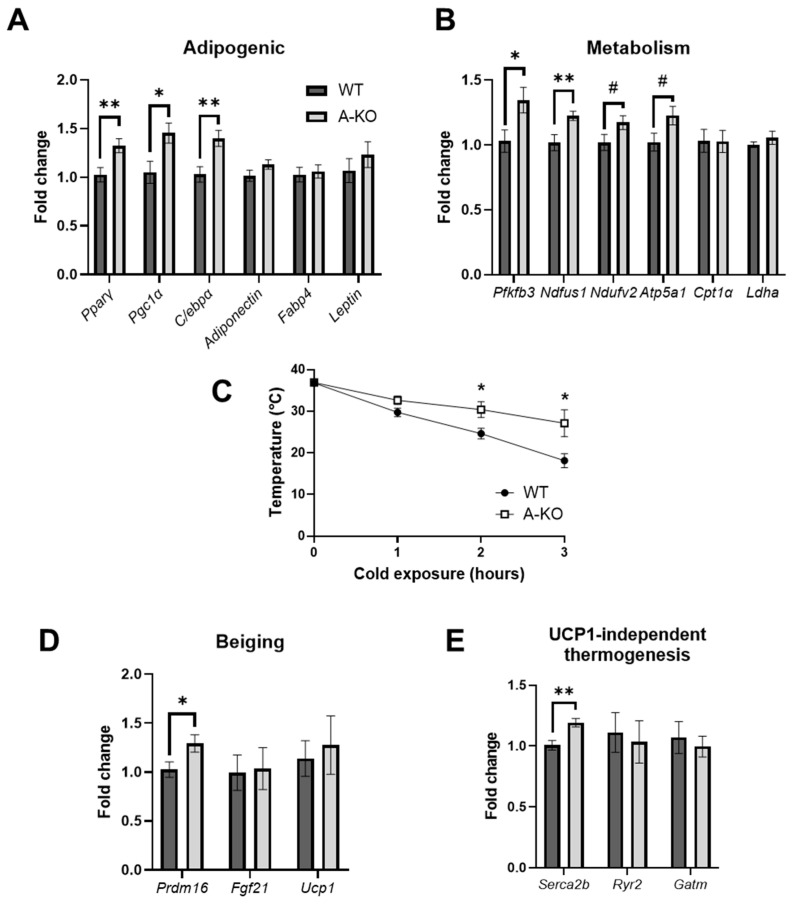
Adipocyte-specific *HDAC9* gene deletion increases expression of adipogenic differentiation and metabolism-associated genes and enhances adaptive thermogenesis in female mice. mRNA expression level of (**A**) adipogenic and (**B**) metabolism genes in SQ of HFD-fed female mice. (**C**) Hourly body temperature measurements during 4 °C acute cold challenge (*n* = 4). mRNA expression level of (**D**) classical beiging and (**E**) UCP-1 independent thermogenesis associated genes in SQ of HFD-fed female mice. Data represent mean ± SEM. * *p* < 0.05; ** *p* < 0.01, # *p* < 0.08. SQ, subcutaneous adipose tissue; PPARɣ, peroxisome proliferator-activated receptor gamma; Pgc1ɑ, PPARɣ coactivator 1-alpha; C/ebpɑ, CCAAT/enhancer-binding protein alpha; Fabp4, fatty acid binding protein 4; Pfkfb3, 6-phosphofructo-2-kinase/fructose-2,6-bisphosphatase 3; Ndfus1, NADH-ubiquinone oxidoreductase; Ndufv2, NADH-ubiquinone oxidoreductase flavoprotein 2; Atp5a1, ATP synthase F1 subunit alpha; Cpt1a, carnitine palmitoyltransferase 1a; Ldha, lactate dehydrogenase A; Prdm16, PR/SET domain 16; Fgf21, fibroblast growth factor 21; Ucp1, uncoupling protein 1; Serca2b, sarco/endoplasmic reticulum Ca^2+^ ATPase 2b; Ryr2, ryanodine receptor 2; Gatm, glycine amidinotransferase.

We previously demonstrated that global *HDAC9* gene deletion promoted adipogenic differentiation and beiging [7]. To determine whether similar mechanisms might contribute to the metabolic phenotype observed in female A-KO mice, we evaluated mRNA expression of adipogenic (Figure 5A and Figure S4A) and metabolic (Figure 5B and Figure S4A) genes in adipose tissues from HFD-fed female mice. Consistent with our previous report of HDAC9 as a negative regulator of adipogenic differentiation [2], SQ from HFD-fed A-KO mice had higher expression of certain genes associated with adipogenic differentiation compared to WT mice (Figure 5A). Additionally, SQ from HFD-fed A-KO mice had elevated expression of the rate limiting gene for glycolysis, *Pfkfb3*, and several genes involved in mitochondrial function (Figure 5B). Visceral fat from HFD-fed A-KO mice exhibited similar trends in gene expression, although the values did not achieve statistical significance (Figure S4).

Next, we subjected approximately 1-year-old HFD-fed female A-KO and WT mice to acute 4 °C cold exposure. Interestingly, A-KO mice were better able to maintain their body temperature following 3 h of cold exposure, despite no difference in basal temperature (Figure 5C). White adipocytes can undergo beiging and contribute to thermogenesis by increasing the expression of genes classically associated with brown adipose tissue, such as uncoupling protein 1 (UCP1). We evaluated the expression of genes classically associated with beiging in adipose tissues from HFD-fed female mice. Interestingly, only *Prdm16*, a positive transcriptional regulator of beiging that has been shown to beneficially alter metabolic health and systemic energy expenditure [10,11], was significantly elevated in SQ from HFD-fed A-KO mice (Figure 5D). Next, we evaluated expression of genes involved in UCP1-independent thermogenesis genes and identified a marginal but significant increase in the expression of *Serca2b*, a protein associated with the Ca^2+^ cycling, in SQ adipose tissue from A-KO mice (Figure 5E). These results suggest that deletion of adipocyte *HDAC9* in female mice increases adipogenic differentiation and adaptive thermogenic capacity in the context of DIO.

### 3.8. Adipose Tissue HDAC9 Is Differentially Regulated in a Sex-Dependent Manner

**Figure 6:**
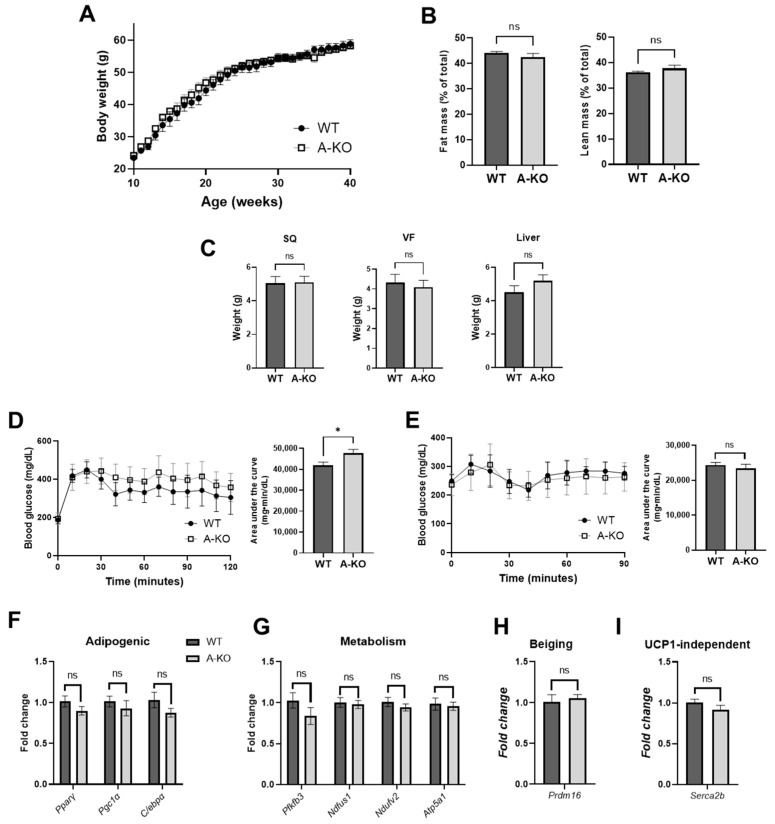
Adipocyte-specific *HDAC9* gene deletion does not improve metabolic health in HFD-fed male mice. (**A**) Growth curves of HFD-fed male WT and A-KO mice (*n* = 11–15). (**B**) Fat mass and lean mass measured by whole body composition nuclear magnetic resonance (*n* = 10–12). (**C**) Tissue weight measurements of SQ, VF and liver at time of sacrifice (*n* = 10–12). Glucose tolerance (**D**) and insulin tolerance test (**E**) quantified by area under the curve (*n* = 4–5). mRNA expression of (**F**) adipogenic, (**G**) metabolic, (**H**) classic beiging and (**I**) UCP1-independent beiging genes in SQ from HFD-fed male mice (*n* = 8). Data represent mean ± SEM. * *p* < 0.05, ns, not significant. SQ, subcutaneous adipose tissue; VF, visceral adipose tissue; PPARɣ, peroxisome proliferator-activated receptor gamma; Pgc1ɑ, PPARɣ coactivator 1-alpha; C/ebpɑ, CCAAT/enhancer-binding protein alpha; Pfkfb3, 6-phosphofructo-2-kinase/fructose-2,6-bisphosphatase 3; Ndfus1, NADH-ubiquinone oxidoreductase; Ndufv2, NADH-ubiquinone oxidoreductase flavoprotein 2; Atp5a1, ATP synthase F1 subunit alpha; Prdm16, PR/SET domain 16; Serca2b; sarco/endoplasmic reticulum Ca^2+^ ATPase 2b.

Accumulating data suggest that adipose tissue function and fat distribution are differentially affected by obese conditions in males and females [12]. Unexpectedly, in contrast to the female mice, HFD-fed male A-KO mice did not exhibit protection against HFD-induced weight gain (Figure 6A), adiposity (Figure 6B,C), glucose intolerance (Figure 6D) or insulin resistance (Figure 6E) compared to HFD-fed male WT mice, despite successful knockdown of *HDAC9* expression (Figure 2A,B). Additionally, A-KO of *HDAC9* in male HFD-fed mice did not alter the expression of adipogenic differentiation (Figure 6F), metabolic (Figure 6G) or beiging genes (Figure 6H) that were elevated in female A-KO mice (Figure 6F–I). Expression of Serca2b, an Ucp1-independent gene, was likewise unaffected in the male A-KO mice (Figure 6I). These contrasting results between male and female mice suggest a sex-dependent effect of adipocyte HDAC9 in obesity.

**Figure 7:**
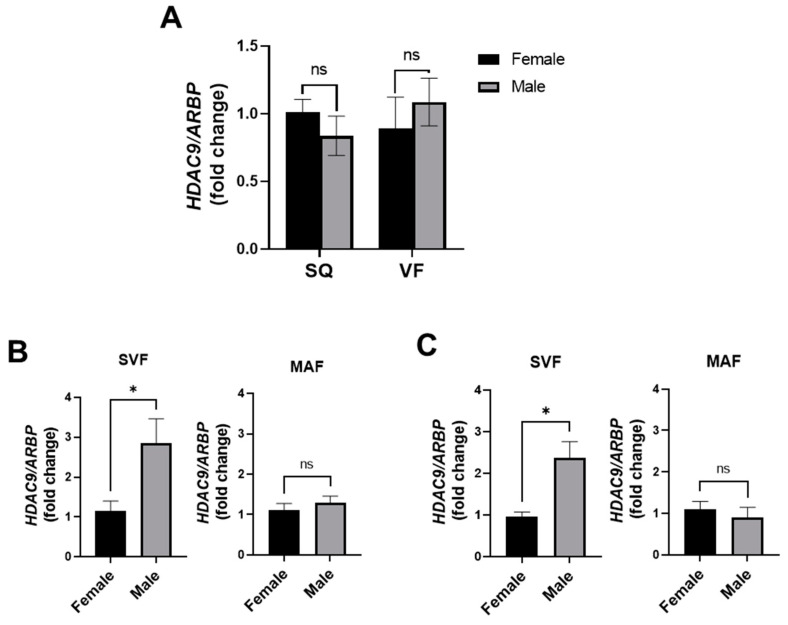
*HDAC9* is differentially expressed in SVF of male compared to female mice. (**A**) *HDAC9* mRNA expression in SQ and VF from HFD-fed female and male WT mice (*n* = 4–7). *HDAC9* mRNA expression in SVF and MAF from SQ (**B**) and VF (**C**) of HFD-fed WT mice (*n* = 6–7). Data represent mean ± SEM. * *p* < 0.05, ns, not significant. SQ, subcutaneous adipose tissue; VF, visceral adipose tissue; SVF, stromovascular fraction; MAF, mature adipocyte fraction.

To gain mechanistic insight into sex differences of HDAC9 in adipose tissue depots, we first compared *HDAC9* gene expression in whole SQ and VF adipose tissues and found no significant difference in *HDAC9* expression between HFD-fed female and male mice (Figure 7A). Next, we fractionated adipose tissues into SVF and MAF and compared *HDAC9* gene expression between DIO male and female mice. While there was no significant difference in the expression level of *HDAC9* in MAF of male versus female mice (Figure 7B,C), male HFD-fed mice had significantly higher *HDAC9* expression (~3-fold) in the SVF of both SQ and VF adipose tissues compared to the female mice (Figure 7B,C). Thus, deletion of *HDAC9* in mature adipocytes is sufficient to protect female, but not male, mice against obesity-related metabolic disease. Upregulated *HDAC9* expression in stromovascular cells of male mice during HFD feeding might have compensated for the deletion of *HDAC9* in mature adipocytes, thus abrogating the beneficial metabolic effects.

## 4. Discussion

Previous studies have demonstrated that HDAC9 is associated with the pathogenesis of cardiometabolic diseases such as atherosclerosis [13,14,15,16,17] and diabetes [7], and that global *HDAC9* KO mice are protected against obesity-related metabolic disease. In this study, we investigated the impact of adipocyte-specific *HDAC9* gene deletion on metabolic function in HFD-induced obesity using both female and male mice. Major findings of this study are (1) adipose tissue *HDAC9* expression was increased in an HFD-induced mouse model of obesity and positively correlated with BMI in humans, (2) in female HFD-fed mice, adipocyte-specific *HDAC9* gene deletion led to reduced weight gain and adipocyte hypertrophy, and improvements in glucose tolerance and insulin sensitivity, despite similar locomotor activity and food consumption. Furthermore, these mice exhibited increased energy expenditure and improved adaptive thermogenic capacity in response to cold exposure, in conjunction with increased expression of adipogenic differentiation, metabolism and beiging-associated genes, (3) unexpectedly, adipocyte-specific *HDAC9* KO did not confer the same benefits to male mice. (4) Mechanistically, we found that HFD-fed male mice had higher *HDAC9* gene expression in the SVF compared to female mice, which may have offset the effects of adipocyte-specific *HDAC9* gene deletion in male mice. Taken together, these data suggest that adipocyte-expressed HDAC9 is detrimental to adipocyte function in obese female mice and provide novel evidence of sex-related differences in HDAC9 cellular expression and contribution to obesity.

In the present study, female A-KO mice exhibited reduced weight gain and improved metabolic function during DIO. However, the beneficial phenotype of A-KO mice was somewhat delayed and milder relative to data previously reported with the global *HDAC9* KO male mice [7]. This is likely explained in part by the fact that *HDAC9* expressed in other cell types (i.e., preadipocytes, inflammatory cells) in adipose tissues may contribute to DIO. Interestingly, in the present study, we observed that *HDAC9* expression was significantly and unexpectedly higher in mature adipocytes compared to SVF (Figure 1B,C), in contrast to the finding of our previous study [7]. The reason for this finding is unclear. In the present study, we used middle age mice (around one-year-old at end of study, a time at which CD-fed mice are typically approaching their heaviest body weight) [18], whereas in our prior study, we studied younger mice (20 weeks old at the termination of the study). Several studies have implicated HDAC family members in aging processes [19,20], and HDAC inhibitors have been proposed as anti-aging therapies [21,22,23]. Thus, the specific impact of aging (independent of obesity) on cellular distribution of HDAC9 expression in adipose tissues remain to be determined. Additionally, our data from human subcutaneous adipose tissues show a positive correlation between BMI and *HDAC9* mRNA expression. However, a more thorough study with a larger sample size that would allow stratification of biological variables such as sex, age, BMI and disease status would aid our understanding of the role of HDAC9 in human health and disease.

One of the interesting aspects of this study is the differential role of adipose HDAC9 in obesity and metabolic disease in female versus male mice. Sex differences in male and female adipose tissues are further complicated by differences in their response to HFD feeding, which is not currently well understood. In response to HFD, males were reported to have greater weight gain, stronger inflammatory responses and enhanced sensitivity to developing insulin resistance [24], while females exhibited larger adipocytes compared to male mice when studied at the same weight following HFD feeding [25]. Additionally, divergent responses in expression of adiponectin and peroxisome proliferator-activated receptor gamma (PPARγ), which are major regulators of adipocyte function, were observed in males versus females in response to HFD feeding [26,27]. Furthermore, Vasconcelos et al. reported that male rats, as compared to female rats, showed higher expression of proinflammatory markers in SQ adipose tissue after HFD feeding [28]. In contrast, activity of the antioxidant enzyme catalase was higher in adipose tissue of female rats. Notably, adipose tissue consists of 20–40% of precursor cells, including stem cells and preadipocytes [29]. Daily turnover of preadipocytes and adipocytes could be as high as 5%, and obesity markedly stimulates preadipocyte replication [30]. Whether the composition of preadipocytes and mature adipocytes in adipose tissue is different between the sexes, particularly in the setting of DIO, is unknown and requires in-depth investigation in the future. Nevertheless, these findings suggest that during DIO, male mice are more susceptible to adipose tissue dysfunction compared to female mice, and, accordingly, interventions directed towards ameliorating obesity-related metabolic disease may have differing efficacy in male versus female mice.

Levels of sex steroid hormones are altered by obesity and can signal to receptors in adipose tissue to alter energy balance [31]. Sex differences in body fat distribution, insulin signaling and lipid metabolism during normal growth and in response to hormonal or nutritional imbalance are also mediated at least in part through sex hormones and the sex chromosome. Our data show that male A-KO mice were not protected against HFD-induced weight gain, suggesting that *HDAC9* expression in other cell types, such as preadipocytes or inflammatory cells recruited during HFD feeding, may contribute to adipose tissue dysfunction and metabolic disease preferentially in male mice. Indeed, we observed increased expression of *HDAC9* in stromovascular cells of obese male mice. Many different cell types are contained within the SVF, and the composition of the SVF differs between male and female tissues. The predominant cell types within the SVF from both male and female adipose tissues are adipose stem cells and preadipocytes. However, SVF from male mice contains a high percentage of leukocytes and CD34+ adipose stem-like cells compared to female [32]. The expression level of *HDAC9* in various cell types in the SVF, and the role of sex hormones in mediating the cell-specific expression of HDAC9, remains to be determined. Crosstalk between preadipocytes, immune cells and adipocytes has also been shown to regulate adipose tissue inflammation and function [33,34,35,36]. Thus, a dynamic interplay between HDAC9 expressing cells within the SVF and adipocytes may contribute to the development of adipose tissue dysfunction and metabolic disease. Future studies are required to investigate this hypothesis.

In conclusion, our findings suggest that HDAC9 expressed in mature adipocytes during DIO plays an important role in regulating adipose tissue function and metabolic disease in female, but not male, mice. Importantly, adipocyte-specific *HDAC9* gene deletion is sufficient to protect against weight gain, adipocyte hypertrophy and metabolic dysfunction in female mice. Obese male mice exhibit much higher levels of *HDAC9* expression in stromovascular cells as compared to female mice, which may have partially compensated for the loss of HDAC9 in mature adipocytes. These results suggest that HDAC9 expressed in adipocytes is detrimental to obesity in female mice and provides novel evidence of sex-related differences in HDAC9 cellular expression that may be important in obesity-related metabolic disease.

## Data Availability

The data presented in this study are available within the article or Supplementary Material.

## Supplementary Materials

The following supporting information can be downloaded at https://www.mdpi.com/article/10.3390/cells11172698/s1. Figure S1: Validation of adipose fractionation; Figure S2: A-KO does not improve metabolic health in CD-fed female mice; Figure S3: A-KO does not improve metabolic health in CD-fed male mice; Figure S4: Adipocyte-specific *HDAC9* gene deletion does not significantly alter gene expression in VF from HFD-fed female mice; Table S1: Demographic data of human subjects; Table S2: Primer sequences.

## References

[1] Choe S.S., Huh J.Y., Hwang I.J., Kim J.I., Kim J.B. (2016). Adipose Tissue Remodeling: Its Role in Energy Metabolism and Metabolic Disorders. Front. Endocrinol..

[2] Chatterjee T.K., Idelman G., Blanco V., Blomkalns A.L., Piegore M.G., Weintraub D.S., Kumar S., Rajsheker S., Manka D., Rudich S.M. (2011). Histone Deacetylase 9 Is a Negative Regulator of Adipogenic Differentiation*. J. Biol. Chem..

[3] Yiew N.K.H., Greenway C., Zarzour A., Ahmadieh S., Goo B., Kim D., Benson T.W., Ogbi M., Tang Y., Chen W. (2019). Enhancer of zeste homolog 2 (EZH2) regulates adipocyte lipid metabolism independent of adipogenic differentiation: Role of apolipoprotein E. J. Biol. Chem..

[4] Chatterjee T.K., Stoll L.L., Denning G.M., Harrelson A., Blomkalns A.L., Idelman G., Rothenberg F.G., Neltner B., Romig-Martin S.A., Dickson E.W. (2009). Proinflammatory Phenotype of Perivascular Adipocytes: Influence of high-fat feeding. Circ. Res..

[5] Yiew N.K.H., Chatterjee T.K., Tang Y., Pellenberg R., Stansfield B.K., Bagi Z., Fulton D.J., Stepp D.W., Chen W., Patel V. (2017). A novel role for the Wnt inhibitor APCDD1 in adipocyte differentiation: Implications for diet-induced obesity. J. Biol. Chem..

[6] Chatterjee T.K., Aronow B.J., Tong W.S., Manka D., Tang Y., Bogdanov V.Y., Unruh D., Blomkalns A.L., Piegore M.G., Weintraub D.S. (2013). Human coronary artery perivascular adipocytes overexpress genes responsible for regulating vascular morphology, inflammation, and hemostasis. Physiol. Genom..

[7] Chatterjee T.K., Basford J.E., Knoll E., Tong W.S., Blanco V., Blomkalns A.L., Rudich S., Lentsch A.B., Hui D.Y., Weintraub N.L. (2013). HDAC9 Knockout Mice Are Protected From Adipose Tissue Dysfunction and Systemic Metabolic Disease During High-Fat Feeding. Diabetes.

[8] Benson T.W., Weintraub D.S., Crowe M., Yiew N.K., Popoola O., Pillai A., Joseph J., Archer K., Greenway C., Chatterjee T.K. (2018). Deletion of the Duffy antigen receptor for chemokines (DARC) promotes insulin resistance and adipose tissue inflammation during high fat feeding. Mol. Cell. Endocrinol..

[9] Schindelin J., Arganda-Carreras I., Frise E., Kaynig V., Longair M., Pietzsch T., Preibisch S., Rueden C., Saalfeld S., Schmid B. (2012). Fiji: An open-source platform for biological-image analysis. Nat. Methods.

[10] Ikeda K., Kang Q., Yoneshiro T., Camporez J.P., Maki H., Homma M., Shinoda K., Chen Y., Lu X., Maretich P. (2017). UCP1-independent signaling involving SERCA2b-mediated calcium cycling regulates beige fat thermogenesis and systemic glucose homeostasis. Nat. Med..

[11] Hasegawa Y., Ikeda K., Chen Y., Alba D.L., Stifler D., Shinoda K., Hosono T., Maretich P., Yang Y., Ishigaki Y. (2018). Repression of Adipose Tissue Fibrosis through a PRDM16-GTF2IRD1 Complex Improves Systemic Glucose Homeostasis. Cell Metab..

[12] Chang E., Varghese M., Singer K. (2018). Gender and Sex Differences in Adipose Tissue. Curr. Diabetes Rep..

[13] Cao Q., Rong S., Repa J., Clair R.S., Parks J.S., Mishra N. (2014). Histone Deacetylase 9 Represses Cholesterol Efflux and Alternatively Activated Macrophages in Atherosclerosis Development. Arter. Thromb. Vasc. Biol..

[14] Lecce L., Xu Y., V’Gangula B., Chandel N., Pothula V., Caudrillier A., Santini M.P., D’Escamard V., Ceholski D.K., Gorski P.A. (2021). Histone deacetylase 9 promotes endothelial-mesenchymal transition and an unfavorable atherosclerotic plaque phenotype. J. Clin. Investig..

[15] Wang M., Gu M., Li Z., Sun B., Cheng X., Dai Z., Li S., Xiao L., Zhao M., Wang Z. (2018). HDAC9 Polymorphisms Predict Susceptibility, Severity, and Short-Term Outcome of Large Artery Atherosclerotic Stroke in Chinese Population. J. Mol. Neurosci..

[16] Qingxu G., Yan Z., Jiannan X., Yunlong L. (2015). Association Between the Gene Polymorphisms of HDAC9 and the Risk of Atherosclerosis and Ischemic Stroke. Pathol. Oncol. Res..

[17] Chiou H.-Y., Bai C.-H., Lien L.-M., Hu C.-J., Jeng J.-S., Tang S.-C., Lin H.-J., Hsieh Y.-C. (2020). Interactive Effects of a Combination of the HDAC3 and HDAC9 Genes with Diabetes Mellitus on the Risk of Ischemic Stroke. Thromb. Haemost..

[18] Petr M.A., Alfaras I., Krawcyzk M., Bair W.-N., Mitchell S.J., Morrell C.H., Studenski S.A., Price N.L., Fishbein K.W., Spencer R.G. (2021). A cross-sectional study of functional and metabolic changes during aging through the lifespan in male mice. eLife.

[19] Yu R., Cao X., Sun L., Zhu J.-Y., Wasko B.M., Liu W., Crutcher E., Liu H., Jo M.C., Qin L. (2021). Inactivating histone deacetylase HDA promotes longevity by mobilizing trehalose metabolism. Nat. Commun..

[20] Moresi V., Williams A.H., Meadows E., Flynn J.M., Potthoff M.J., McAnally J., Shelton J.M., Backs J., Klein W.H., Richardson J.A. (2010). Myogenin and Class II HDACs Control Neurogenic Muscle Atrophy by Inducing E3 Ubiquitin Ligases. Cell.

[21] McIntyre R.L., Daniels E.G., Molenaars M., Houtkooper R.H., Janssens G.E. (2019). From molecular promise to preclinical results: HDAC inhibitors in the race for healthy aging drugs. EMBO Mol. Med..

[22] Gao Z., Yin J., Zhang J., Ward R.E., Martin R.J., Lefevre M., Cefalu W.T., Ye J. (2009). Butyrate Improves Insulin Sensitivity and Increases Energy Expenditure in Mice. Diabetes.

[23] Walsh M.E., Bhattacharya A., Sataranatarajan K., Qaisar R., Sloane L.B., Rahman M.M., Kinter M., Van Remmen H. (2015). The histone deacetylase inhibitor butyrate improves metabolism and reduces muscle atrophy during aging. Aging Cell.

[24] Fuente-Martín E., Argente-Arizón P., Ros P., Argente J., Chowen J.A. (2013). Sex Differences in Adipose Tissue: It Is Not Only a Question of Quantity and Distribution. Adipocyte.

[25] Nickelson K.J., Stromsdorfer K.L., Pickering R., Liu T.-W., Ortinau L.C., Keating A.F., Perfield J.W. (2012). A Comparison of Inflammatory and Oxidative Stress Markers in Adipose Tissue from Weight-Matched Obese Male and Female Mice. Exp. Diabetes Res..

[26] Estrany M.E., Proenza A.M., Lladó I., Gianotti M. (2011). Isocaloric intake of a high-fat diet modifies adiposity and lipid handling in a sex dependent manner in rats. Lipids Heal. Dis..

[27] Estrany M.E., Proenza A.M., Gianotti M., Lladó I. (2012). High-fat diet feeding induces sex-dependent changes in inflammatory and insulin sensitivity profiles of rat adipose tissue. Cell Biochem. Funct..

[28] Vasconcelos R.P., Peixoto M.S., De Oliveira K.A., Ferreira A.C.F., Coelho-De-Souza A.N., Carvalho D., Oliveira A., Fortunato R.S. (2019). Sex differences in subcutaneous adipose tissue redox homeostasis and inflammation markers in control and high-fat diet fed rats. Appl. Physiol. Nutr. Metab..

[29] Hauner H. (2005). Secretory factors from human adipose tissue and their functional role. Proc. Nutr. Soc..

[30] Joe A.W., Yi L., Even Y., Vogl A.W., Rossi F.M. (2009). Depot-Specific Differences in Adipogenic Progenitor Abundance and Proliferative Response to High-Fat Diet. Stem Cells.

[31] Newell-Fugate A.E. (2017). The role of sex steroids in white adipose tissue adipocyte function. Reproduction.

[32] Frazier T., Lee S., Bowles A., Semon J., Bunnell B., Wu X., Gimble J. (2018). Gender and age-related cell compositional differences in C57BL/6 murine adipose tissue stromal vascular fraction. Adipocyte.

[33] Caër C., Rouault C., Le Roy T., Poitou C., Aron-Wisnewsky J., Torcivia A., Bichet J.-C., Clément K., Guerre-Millo M., André S. (2017). Immune cell-derived cytokines contribute to obesity-related inflammation, fibrogenesis and metabolic deregulation in human adipose tissue. Sci. Rep..

[34] Rajbhandari P., Arneson D., Hart S.K., Ahn I.S., Diamante G., Santos L.C., Zaghari N., Feng A.-C., Thomas B.J., Vergnes L. (2019). Single cell analysis reveals immune cell–adipocyte crosstalk regulating the transcription of thermogenic adipocytes. eLife.

[35] Macdougall C.E., Wood E.G., Loschko J., Scagliotti V., Cassidy F.C., Robinson M.E., Feldhahn N., Castellano L., Voisin M.-B., Marelli-Berg F. (2018). Visceral Adipose Tissue Immune Homeostasis Is Regulated by the Crosstalk between Adipocytes and Dendritic Cell Subsets. Cell Metab..

[36] Chazenbalk G., Bertolotto C., Heneidi S., Jumabay M., Trivax B., Aronowitz J., Yoshimura K., Simmons C.F., Dumesic D.A., Azziz R. (2011). Novel Pathway of Adipogenesis through Cross-Talk between Adipose Tissue Macrophages, Adipose Stem Cells and Adipocytes: Evidence of Cell Plasticity. PLoS ONE.

