# Supplementary material for: Sex-Dependent Role of Adipose Tissue HDAC9 in Diet-Induced Obesity and Metabolic Dysfunction
Source: Cells. 2022 Aug 30;11(17):2698. doi: 10.3390/cells11172698 (PMC9454798; doi:10.3390/cells11172698)
Supplement: Supplementary file 1 [file cells-11-02698-s001.zip › cells-1794736-supplementary.pdf]

Supplemental Figure S1

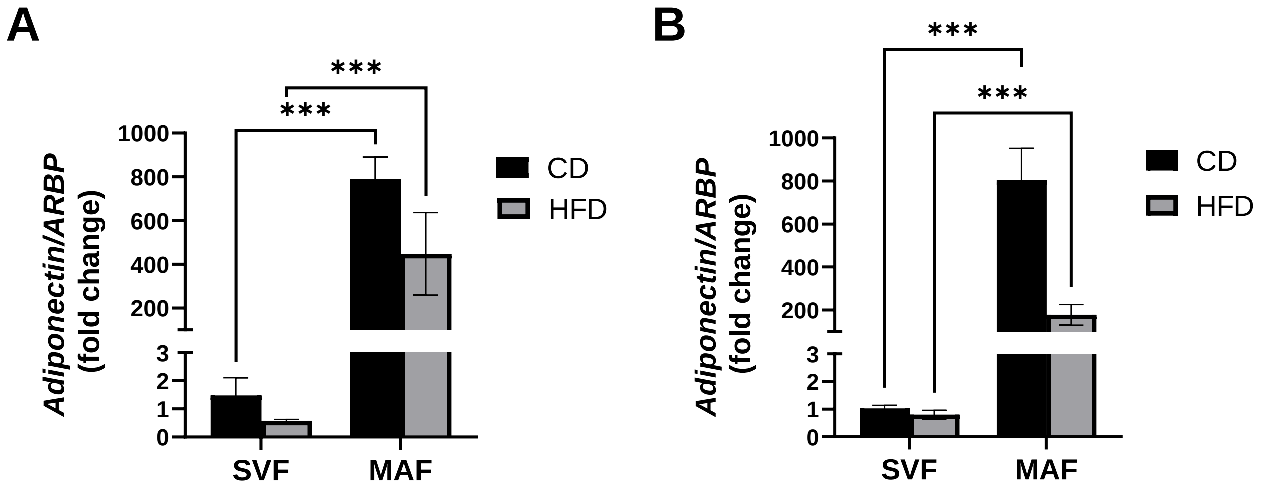

Figure S1. Validation of adipose fractionation. *Adiponectin* mRNA expression in SVF and MAF from SQ **(A)** and VF **(B)** of female WT mice either on CD or HFD (n=5-6). Data represent mean ± SEM. ***p<0.001. SQ, subcutaneous adipose tissue; VF, visceral adipose tissue; CD, chow diet; HFD, high fat diet; SVF, stromovascular fraction; MAF, mature adipocyte fraction.

Supplemental Figure S2

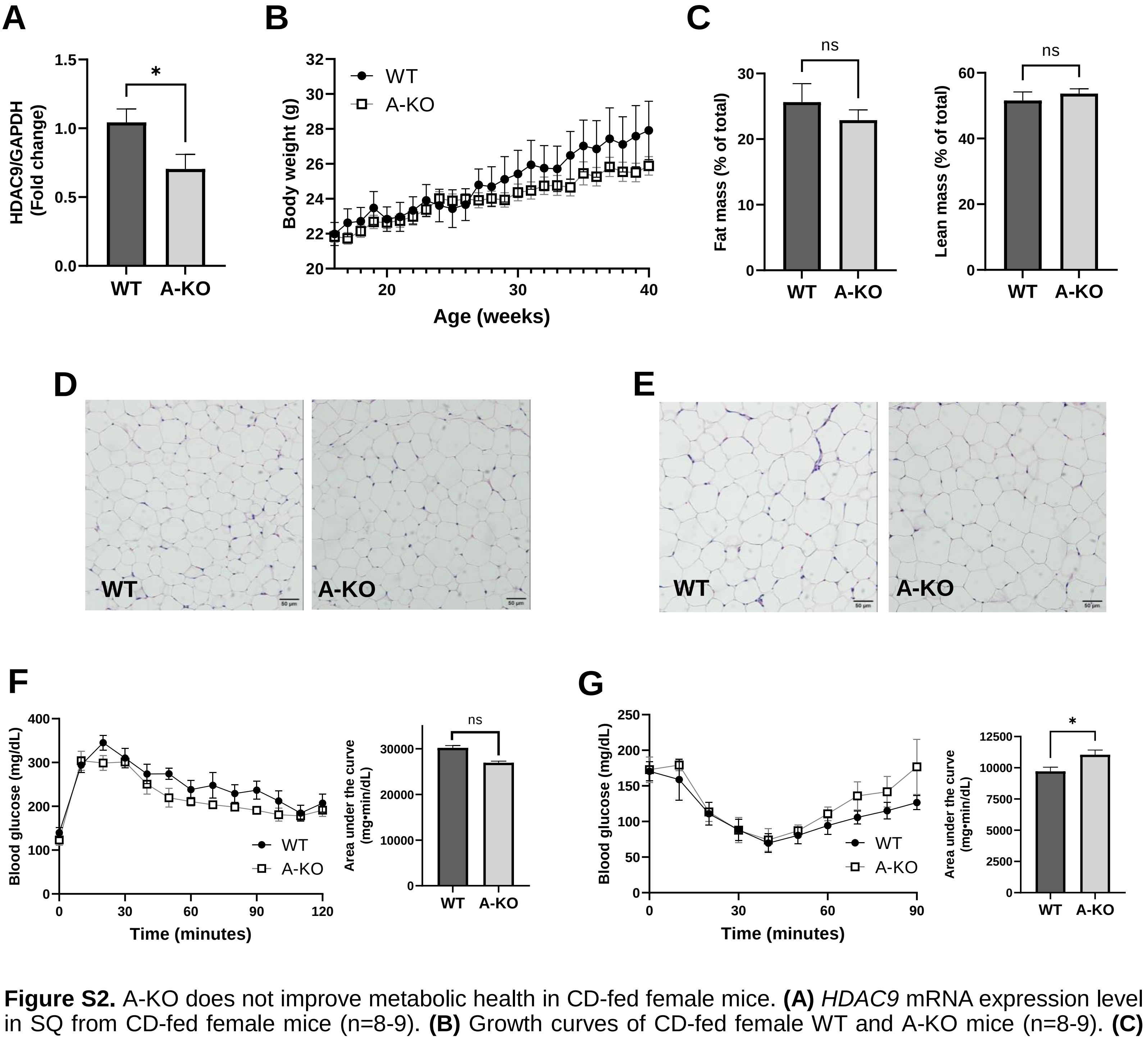

Figure S2. A-KO does not improve metabolic health in CD-fed female mice. **(A)** *HDAC9* mRNA expression level in SQ from CD-fed female mice (n=8-9). **(B)** Growth curves of CD-fed female WT and A-KO mice (n=8-9). **(C)** Fat mass and lean mass measured by whole body composition nuclear magnetic resonance (n=8-9). Representative H&E images of SQ **(D)** and VF **(E)** from CD-fed female WT and A-KO mice. Glucose tolerance **(F)** and insulin tolerance test **(G)** quantified by area under the curve (n=4). Data represent mean ± SEM. *p<0.05. SQ, subcutaneous adipose tissue; VF, visceral adipose tissue.

Supplemental Figure S3

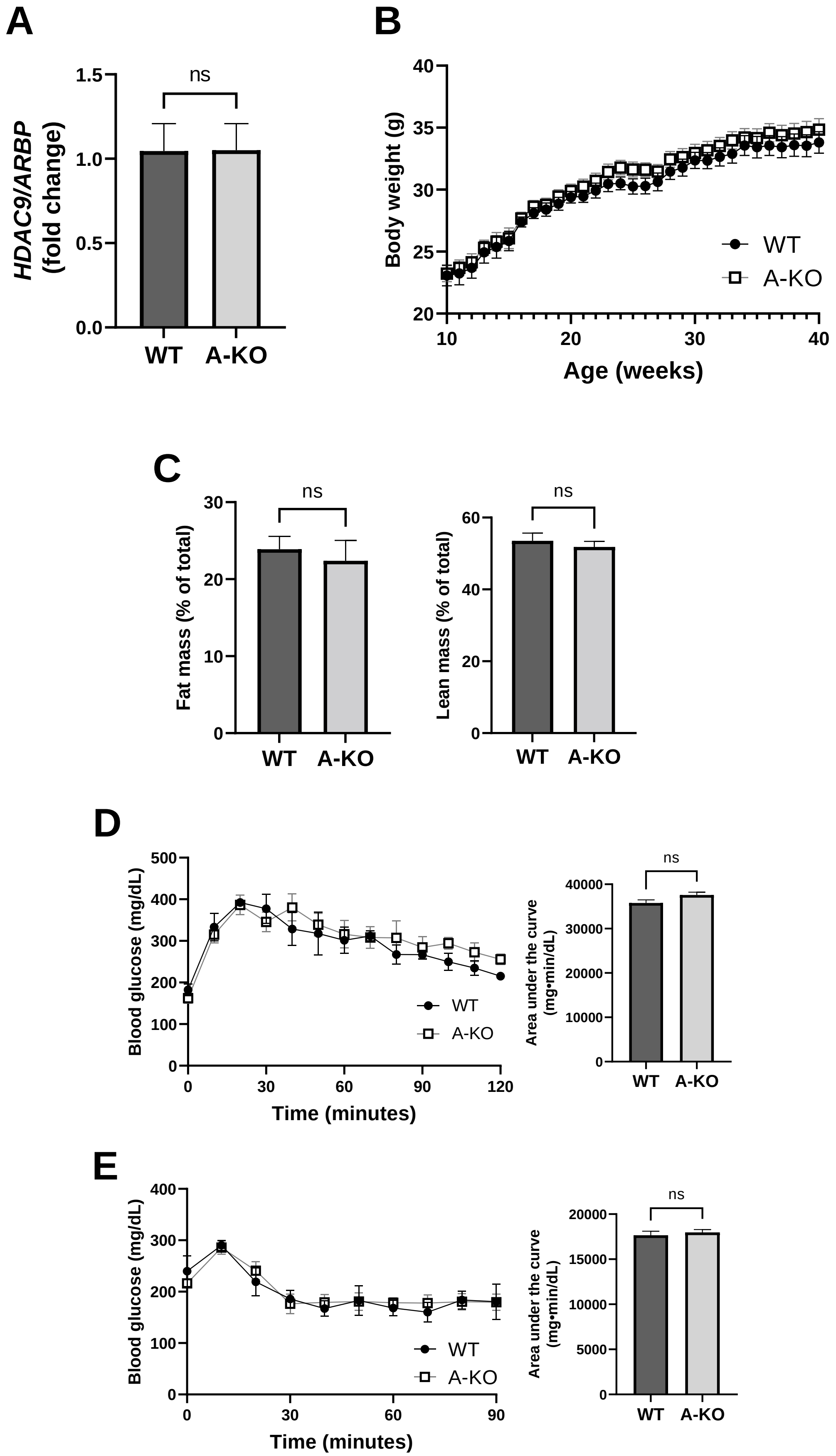

Figure S3. A-KO does not improve metabolic health in CD-fed male mice. **(A)** Expression of *HDAC9* mRNA in SQ CD-fed male mice determine by qPCR (n=3-4). **(B)** Growth curves of CD-fed male WT and A-KO mice (n=10-15). **(C)** Fat mass and lean mass measured by whole body composition nuclear magnetic resonance (n=7-10). Glucose tolerance **(D)** and insulin tolerance test **(E)** quantified by area under the curve (n=4). Data represent mean ± SEM.

Supplemental Figure S4

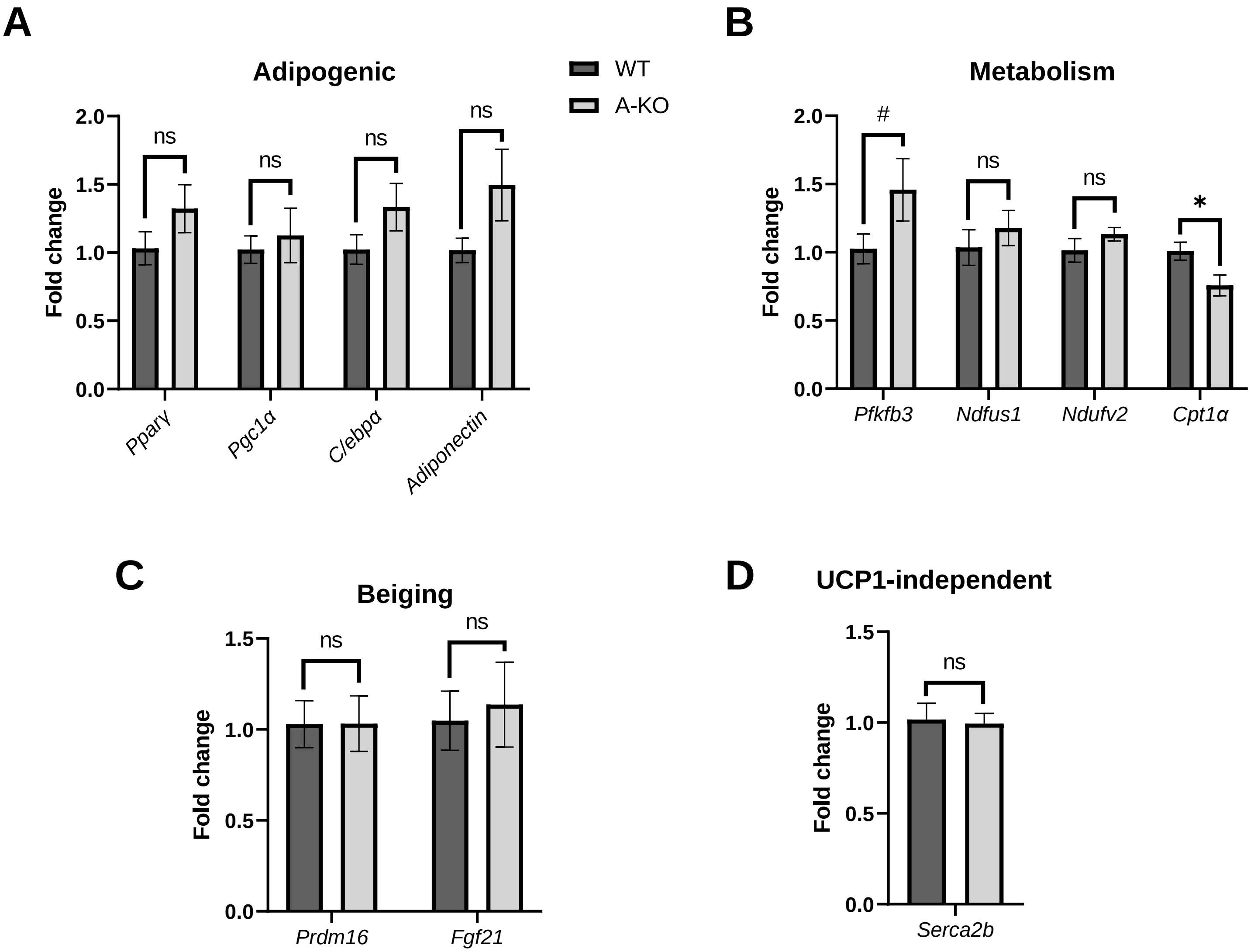

Figure S4. Adipocyte-specific *HDAC9* gene deletion does not significantly alter gene expression in VF from HFD-fed female mice. mRNA expression of **(A)** adipogenic, **(B)** metabolic genes **(C)** classic beiging and **(D)** UCP1-independent beiging genes in SQ from HFD-fed female mice (n=5-6). Data represent mean ± SEM. $^{\#}p<0.1$; $^{*}p<0.05$. VF, visceral adipose tissue; PPARɣ, peroxisome proliferator-activated receptor gamma; Pgc1α, PPARɣ coactivator 1-alpha; C/ebpα, CCAAT/enhancer-binding protein alpha; Pfkfb3, 6-phosphofructo-2-kinase/fructose-2,6-bisphosphatase 3; Ndfus1, NADH-ubiquinone oxidore-ductase; Ndufv2, NADH-ubiquinone oxidoreductase flavoprotein 2; Cpt1a, carnitine palmitoyltransferase 1a; Prdm16, PR/SET domain 16; Fgf21, fibroblast growth factor 21; Serca2b, sarco/endoplasmic reticulum Ca2+ ATPase 2b.

Supplemental Table S1

Table S1: Demographic data of human subjects.

BMI (kg/m2)	Age (years)	Gender	Diabetes Mellitus
21.4	55	M	Yes
23.3	50	M	No
24.0	68	M	No
24.0	43	M	Yes
24.5	64	M	No
25.0	62	M	No
27.1	65	M	No
27.8	60	M	No
28.7	82	M	No
34.9	53	F	No
35.3	44	M	No
42.2	64	F	Yes

Supplemental Table S2

Table S2: Primer Sequences.

Gene Name	Species	Forward	Reverse
Adiponectin	mouse	GCACTGGCAAGTTCTACTGCAACA	AGAGAACGGCCTTGTCCTTGTTGA
Arbp	mouse	AGCTGAAGCAAAGGAAGAGTCGGA	ACTTGGTTGCTTTGGCGGGATTAG
Aromatase	mouse	GCTGAACCCCATGCAGTATAA	AGCCAAAAGGCTGAAAGTACC
Atp5a1	mouse	TCTCCATGCCTCTAACACTCG	CCAGGTCAACAGACGTGTCAG
*C/ebp*ɑ	mouse	AGAAGTCGGTGGACAAGAACAGCA	GCGTTGTTTGGCTTTATCTCGGCT
*Cpt1*ɑ	mouse	CATGATTGCAAAGATCAATCGG	CTTGACATGCGGCCAGTG
Fabp4	mouse	ATGAAATCACCGCAGACGACAGGA	TGTGGTCGACTTTCCATCCCACTT
Fgf21	mouse	CTCCACATGAGAGCTAGAACAC	CTGATATCCAGGGCGAAGTAAG
Gatm	mouse	CAGACACAAATTGGCCGCTC	CCCAGGTAGTTTGTAACCTGGC
Hdac9	mouse	AGGATGATGATGCCTGTGGTGGAT	GAGTTGTGCTTGATGCTGCCTTGT
Ldha	mouse	ACATTGTCAAGTACAGTCCACAC	TTCCAATTACTCGGTTTTTGGGA
Leptin	mouse	CAAGCAGTGCCTATCCAGA	AAGCCCAGGAATGAAGTCCA
Ndfus1	mouse	AACCACTGTTCTGCAGGCTT	AGCGACAACCTTTGGAGCTT
Ndfuv2	mouse	GCAAGGAATTTGCATAAGACAGC	TAGCCATCCATTCTGCCTTTG
Pfkfb3	mouse	TACGAAGCTCACACGCTATCT	GCCTCTCGTCGATACTGACCTA
*Pgc1*ɑ	mouse	CCCTGCCATTGTTAAGACC	TGCTGCTGTTCCTGTTTTC
*Ppar*ɣ	mouse	ACATAAAGTCCTTCCCGCTGACCA	AAATTCGGATGGCCACCTCTTTGC
Prdm16	mouse	AGATGAACCAGGCATCCACTCGAA	ACTTCCCGGCTAAGCTGTCATCAT
Ryr2	mouse	ATGGCTTTAAGGCACAGCG	ATGGCTTTAAGGCACAGCG
Serca2b	mouse	ACCTTTGCCGCTCATTTTCCAG	AGGCTGCACACACTCTTTACC
Tst	mouse	CCTGCTGTAGGTTCACCTTTTAGG	GGAGGCACCAAGAGCAATTCTAAA
Ucp1	mouse	AAGCTGTGCGATGTCCATGT	AAGCCACAAACCCTTTGAAAA
Gapdh	human	CGACTTCAACAGCAACTCCCACTCTTCC	TGGGTGGTCCAGGGTTTCTTACTCCTT
Hdac9	human	TACTCAGGCGGAAGGATGGA	CCAGTTGGCCCATTGTTTGG
